# Efficiency, cytotoxicity, and survivability evaluation of *Salmonella* phage cocktail against *Salmonella* derived from broiler sources

**DOI:** 10.14202/vetworld.2025.475-483

**Published:** 2025-02-26

**Authors:** Wattana Pelyuntha, Thamonwan Narkpao, David Yembilla Yamik, Pichamon Kiatwuthinon, Arsooth Sanguankiat, Attawit Kovitvadhi, Kitiya Vongkamjan

**Affiliations:** 1 Futuristic Science Research Center, School of Science, Walailak University, Thasala, Nakhon Si Thammarat 80160, Thailand; 2 Research Center for Theoretical Simulation and Applied Research in Bioscience and Sensing, Walailak University, Thasala, Nakhon Si Thammarat 80160, Thailand; 3 Department of Biotechnology, Faculty of Agro-Industry, Kasetsart University, Chatuchak, Bangkok 10900, Thailand; 4 Department of Biochemistry, Faculty of Science, Kasetsart University, Chatuchak, Bangkok 10900, Thailand; 5 Department of Veterinary Public Health, Faculty of Veterinary Medicine, Kasetsart University, Kamphaeng Saen, Nakhon Pathom 73140, Thailand; 6 Department of Physiology, Faculty of Veterinary Medicine, Kasetsart University, Bangkok, 10900, Thailand

**Keywords:** antibiotic resistance, bacteriophage therapy, food safety, phage cocktail, poultry production, *Salmonella*

## Abstract

**Background and Aim:**

*Salmonella* is a leading cause of foodborne illnesses worldwide, often linked to poultry products. Antibiotic resistance among *Salmonella* strains has increased the need for alternative decontamination strategies, such as bacteriophage (phage) therapy. This study evaluates the lytic efficiency, cytotoxicity, and survivability of a *Salmonella* phage cocktail derived from wastewater sources.

**Materials and Methods:**

A total of 251 *Salmonella enterica* isolates from broiler production chains were tested against two selected phages (WP109 and WP128). The phages were characterized for lytic ability, cytotoxicity on Caco-2 cells, and survivability under simulated gastrointestinal and harsh environmental conditions. A cocktail of the phages was further tested for efficiency at different multiplicities of infection (MOIs) against representative *Salmonella* strains.

**Results:**

Phage WP109 lysed 91.2% of *Salmonella* isolates, while WP128 lysed 78.2%. The phage cocktail exhibited a significant reduction of *Salmonella* counts at MOI 10^4^, achieving up to a 4.4 log CFU/mL reduction *in vitro*. The cocktail maintained 99.9% survivability in simulated gastric conditions and displayed no cytotoxic effects on Caco-2 cells. Moreover, it was resistant to various ionic sanitizers and pH levels ranging from 2 to 11.

**Conclusion:**

The developed phage cocktail demonstrated high lytic efficacy, stability, and safety under simulated conditions, highlighting its potential as a biocontrol agent in the broiler production chain. These findings support its application in reducing Salmonella contamination while addressing the challenges posed by antibiotic resistance.

## INTRODUCTION

*Salmonella* is one of the most common foodborne bacteria associated with human infection worldwide. According to Majowicz *et al*. [1], *Salmonella* accounts for more than 9 million cases of gastrointestinal infection annually, resulting in 155,000 deaths worldwide [1]. Most *Salmonella* infections are due to the consumption of *Salmonella*-contaminated food. Broilers and broiler products constitute a major component of the human diet but are often at risk of *Salmonella* contamination, which can lead to salmonellosis. Animals, especially broilers and poultry in general, serve as primary reservoirs of *Salmonella*, making broilers and poultry products significant contributors to outbreaks [2, 3]. Many researchers have reported the incidence of *Salmonella* contamination in the broiler production chain, from farm to fork, including at processing plants, where cross-contamination often leads to infections [2–6]. Although antibiotics can deactivate these bacteria, the recent emergence of antibiotic-resistant *Salmonella* strains has made this approach challenging and sometimes ineffective [7, 8]. This poses a global public health concern and causes significant economic losses, emphasizing the need for alternative decontamination and treatment strategies. Phages are promising alternatives to antibiotics. They are viruses that infect and replicate in bacteria using the bacterial DNA replication machinery, killing the bacteria. They are also self-amplifying in nature and can evolve through mutations to overcome bacterial defense mechanisms, thereby maintaining their effectiveness compared with antibiotics. They can lyse bacterial pathogens from various sources, including food-producing animals and poultry [9–11]. For instance, *Salmonella* phages have been reported to lyse over 96% of *Salmonella* in chick feces [12]. Hosny *et al*. [13] also reported the 100% lytic ability of a *Salmonella* phage on *Salmonella enterica* from commercial broiler litters in Egypt. Previous studies by [14, 15] have further demonstrated the effectiveness of phages in lysing antibiotic-resistant and multidrug-resistant bacterial strains. For example, ciprofloxacin-resistant *Salmonella* was reduced by 33.3%–93.3% [16]. These findings highlight the potential of phages as alternatives to antibiotics.

Although phages have been proven effective, their efficiency and suitability for controlling bacterial contamination or infections in the animal production chain could be hindered by several factors, including their host range, survivability under various conditions, and cytotoxic effects on host cells. Studies have shown that while some strains of phages exhibit narrow host ranges by lysing only specific bacterial species, others display broader lytic abilities [17, 18]. The host range of phages varies depending on the specific phage strain. For instance, the host range of the *Salmonella* phage E4, as determined by Torkashvand *et al*. [19], exhibited effective deactivation of various motile and non-motile *S. enterica* serovars. A phage cocktail (a combination of two or more phages) increases the efficiency and likelihood of lysing many strains of bacteria [20]. However, this effect could be affected by other conditions under which the phage cocktail is applied. For instance, phages are often applied under various conditions (e.g., pH, temperature, ion concentration, and salinity levels in the animal gut) and in combination with sanitizers, which can influence their survival. Many studies have reported the survivability of phages under various harsh conditions [20–24] and the safety of phages and phage cocktails in host cells [17, 19]. However, this may depend on the specific phage strain. Although phage cocktails can serve as effective alternatives for controlling bacterial infections and contamination in the broiler production chain, their potential cytotoxicity toward host cells may pose a barrier to their implementation.

These findings underscore the need for innovative strategies to address these challenges and achieve better outcomes. This study aims to evaluate the efficiency, survivability, and cytotoxicity of *Salmonella* phage cocktail for its potential application as a biocontrol agent in the broiler production chain. Specifically, it investigates the lytic ability of phages WP109 and WP128 against *Salmonella* isolates, their survivability under simulated gastrointestinal and harsh environmental conditions, and their compatibility with ionic sanitizers. The overarching goal is to explore a sustainable alternative to antibiotics for reducing *Salmonella* contamination in poultry production, addressing public health and food safety concerns associated with antibiotic resistance.

## MATERIALS AND METHODS

### Ethical approval

Humans and animals were not included in this study. Therefore, ethical approval was not required.

### Study period and location

The study was conducted from October 2021 to September 2022 at the Department of Biotechnology, Faculty of Agro-Industry, Kasetsart University, Bangkok, Thailand.

### Bacterial strains and culture conditions

A total of 251 strains of *S. enterica* were received from the Department of Veterinary Public Health, Faculty of Veterinary Medicine, Kasetsart University. All strains were previously isolated from broiler farms (B), grandparent (G), and parent (P) stock farms, broiler processing plants (SL and SP), hatcheries (H), and feed factories (F). All strains were kept in glycerol solution at −20°C and stored at the Department of Biotechnology, Faculty of Agro-Industry, Kasetsart University. The strains were pre-cultured in tryptic soy broth (HiMedia Laboratories, India) at 37°C overnight before further studies.

### Phage lysate preparation

Two *Salmonella* phages, including vB_SenS_WP109 (WP109) and vB_SenP_WP128 (WP128) from our collection, were chosen for this study. These phages were initially recovered from wastewater collected at a wastewater treatment station. The criteria for phage selection were based on the lytic patterns of the two phages against multidrug-resistant and foodborne outbreak-related isolates of *Salmonella* obtained from the broiler production chain in Eastern and Southern Thailand [15]. These phages demonstrated high lytic ability, with a lytic coverage of 88.9%–77.8%, respectively, against the *Salmonella* species used in our previous study by Pelyuntha *et al*. [15]. *Salmonella* Kentucky S1H28 was used as a natural host for the propagation of phage WP109, whereas *Salmonella* Agona H2-016 was the host for phage WP128 [15]. A double-layer agar assay and phage suspension harvesting were performed using salt magnesium buffer. Filtrates were collected after passing through 0.20 μm syringe filters and were stored at 4°C until analysis [15, 16, 25]. Phage titers were enumerated by observing plaques on each plate containing the given dilutions of *S*. Kentucky S1H28 host.

### Determination of phage lytic ability

The lytic abilities of the two phages were investigated by dropping 20 μL of phage lysate (8 log plaque-forming unit (PFU)/mL) onto lawn cultures of given *S*. *enterica* isolates prepared on an overlay. All plates were observed for the appearance of the lysis zone after incubation at 37°C for 18 h [15, 26].

### Phage cocktail preparation and *in vitro* efficiency evaluation

The phages were mixed in equivalent proportions (a ratio of 1:1) to obtain the working cocktail stock. The efficacy of the developed phage cocktail was investigated on two representative *S. enterica* strains (*S. enterica* subsp. *enterica* serovar Enteritidis S5-371 and *S. enterica* subsp. *enterica* serovar Typhimurium S5-370). A 50 mL of the bacterial suspension (at a final concentration of 3 log colony forming units/mL) was mixed with 50 mL of the developed phage cocktail (at final concentrations of 5, 6, and 7 log PFU/mL) to achieve an effective multiplicity of infection (MOI) of 10^2^, 10^3^, and 10^4^, respectively. We further incubated these samples at 37°C for 48 h in a shaking incubator (200 rpm). Each culture without a phage cocktail was used as a control. The reduction of *Salmonella* was evaluated every 6 h using a direct plate count on tryptic soy agar plates. The remaining phages were also monitored using the double-layer agar technique.

### Cytotoxicity of the phage cocktail

The cytotoxic effects of the developed phage cocktail on the intestinal cell line, Caco-2 cells HTB-37^™^, were evaluated. Caco-2 cells (8,000 cells/well) were seeded in a 96-well microtiter plate and grown in Eagle’s Minimum Essential Medium (Gibco, Thermo Fisher Scientific, USA) supplemented with 1% (v/v) penicillin/streptomycin and 10% (v/v) fetal bovine serum under a 5% saturated CO_2_ atmosphere at 37°C and 95% humidity for 24 h. The culture medium was then replaced with a serum-free medium containing a phage cocktail at a final concentration of 5–8 log PFU/mL, followed by incubation for 24, 48, and 72 h under the given conditions. The culture fluid of Caco-2 cells was removed. A suspension of 0.5 mg/mL MTT reagent (Sigma Chemical Co., St Louis, MO, USA) was added and incubated for 3 h, followed by the addition of DMSO to dissolve the formazan crystals, and the optical densities were measured at 570 nm [20]. The percentage cell viability was then calculated.

### Phage survivability in simulated chicken gastrointestinal conditions

For survivability under gastric conditions, a phage cocktail of approximately 9 log PFU/mL was added to the simulated gastric fluid containing 0.6 g/mL pepsin (Sigma Aldrich, USA) in 0.2% NaCl solution (pH 2.0), followed by incubation at 37°C for 2 h. The mixture was then transferred to 2% (w/v) bile salt solution and incubated at 37°C for 1 h. To assess survivability under intestinal conditions, simulated intestinal fluid containing 1 g/mL pancreatin (Sigma Aldrich, USA) in 0.2% NaCl solution (pH 8.5) was used to treat the phage cocktail, followed by incubation at 37°C for 4 h. Phage titers were determined using a double-overlay technique [20].

### Phage survivability under harsh conditions

The survivability of the phage cocktail (~9 log PFU/mL) at different temperatures (4, 25, 37, 45, and 60°C) was evaluated after 1, 3, and 24 h of incubation. The effects of acidic and alkaline conditions on the survivability (~9 log PFU/mL) of the phage cocktail were determined at different pH levels (2, 5, 7, 9, and 11) by incubation at ambient temperature for 1, 3, and 24 h. The phage cocktail was treated with 0.1, 0.25, 0.5, 1, 2.5, and 5% (v/v) free chlorine at ambient temperature for 1 and 24 h. All assays were performed in triplicate, and the phage titer was determined using the double-overlay method [27].

### Phage survivability in sanitizers

The survivability of the developed phage cocktail was investigated with different sanitizers. Briefly, a phage cocktail at an approximate concentration of 9 log PFU/mL was reacted with alcohol ethoxylate (non-ionic sanitizer), linear alkylbenzene sulfonic acid (anionic sanitizer), and quaternary ammonium compounds (cationic sanitizer) at different concentrations ranging from 0.1% to 5.0% (v/v) for 1 h at ambient temperature. The phage cocktail with the same concentration of sanitizers was kept as a control suspension. Phage titers were determined using the double-overlay technique.

### Statistical analysis

All experiments were performed in triplicate, and the data are expressed as mean ± standard deviation (SD). Statistical comparisons were conducted using SPSS Statistics software version 22.0 for Windows (IBM Corp., Armonk, NY, USA). Differences between treatment and control groups were analyzed using an independent sample t-test, while multiple group comparisons were evaluated using one-way analysis of variance (ANOVA) followed by Tukey’s *post hoc* test to identify significant differences among means. Phage survivability across different environmental conditions, such as temperature, pH, and sanitizer concentrations, was also assessed using ANOVA, with specific pairwise comparisons performed where applicable. Results were considered statistically significant at a threshold of p < 0.05. Where appropriate, results are presented graphically with statistical annotations (e.g., asterisks indicating significant differences). All statistical tests ensured normality and homoscedasticity assumptions before analysis, with non-parametric tests applied if these assumptions were violated.

## RESULTS

### Determination of phage lytic ability

The two phages WP109 and WP128 demonstrated 100% lytic ability against all the *Salmonella* isolates from the grandparent stock (G) and feed factories (F). Phage WP109 lysed all the *Salmonella* isolates recovered from the parent stock farm (P) and hatcheries (H), whereas phage WP128 lysed the *Salmonella* isolates from sources P and H by 95.1% and 62.3%, respectively. The two phages also lysed the *Salmonella* isolates from the broiler farm (B) and the two broiler processing plants (SL and SP), as shown in Table 1. Overall, WP109 and WP128 lysed 229 and 198 isolates out of the tested *S. enterica* strains, representing 91.2% and 78.2%, respectively.

**Table 1:** Lytic ability of phages WP109 and WP128 on *Salmonella* isolated from broiler sources.

Sources	vB_SenS_WP109	vB_SenP_WP128
Grandparent stock farm (G)	1/1 (100)	1/1 (100)
Feed factories (F)	4/4 (100)	4/4 (100)
Parent stock farm (P)	61/61 (100)	58/61 (95.1)
Hatcheries (H)	41/41 (100)	38/61 (62.3)
Broiler farm (B)	51/58 (87.9)	33/58 (56.9)
Broiler processing plant (SL)	37/38 (97.4)	37/38 (97.4)
Broiler processing plant (SP)	34/48 (70.8)	27/48 (56.3)
Total	229/251 (91.2)	198/251 (78.9)

### *In vitro* efficiency evaluation

Different MOIs of the phage cocktail treatments significantly differed in the reduction of the representative strains of *Salmonella*. For *Salmonella* Enteritidis, the highest decrease was observed at MOI 10^4^ at 6 h post-treatment, as indicated by 3.5 log-unit reduction (p < 0.05), followed by MOI 10^3^ (2.1 log-unit; p < 0.05) and MOI 10^2^ (1.6 log-unit; p < 0.05). At 24 h, the phage cocktail significantly reduced the count of *S*. Enteritidis by 2.5, 3.6, and 4.4 log CFU/mL at MOIs of 10^2^, 10^3^, and 10^4^, respectively (Table 2).

**Table 2:** Efficiency of the developed phage cocktails on representative *Salmonella* strains.

Treatments	Time (h)	Bacterial count (log CFU/mL)*			

		Control	MOI 10^2^	MOI 10^3^	MOI 10^4^
*Salmonella* Enteritidis S5-371	0	3.97 ± 0.02^a^	3.95 ± 0.01^a^	3.91 ± 0.03^a^	3.62 ± 0.01^b^
	6	6.06 ± 0.04^b^	4.44 ± 0.09^b^*	3.94 ± 0.03^a^*	2.59 ± 0.11^a^*
	12	8.91 ± 0.04^c^	6.38 ± 0.06^c^*	5.35 ± 0.03^b^*	3.08 ± 0.04^ab^*
	18	9.59 ± 0.04^d^	8.10 ± 0.05e*	7.11 ± 0.07^d^*	6.31 ± 0.03^d^*
	24	9.83 ± 0.04^d^	7.36 ± 0.04^d^*	6.25 ± 0.02^c^*	5.40 ± 0.03^c^*
*Salmonella* Typhimurium S5-370	0	3.79 ± 0.02^a^	3.95 ± 0.01^a^	3.91 ± 0.03^a^	3.62 ± 0.02^b^
	6	6.43 ± 0.02^b^	4.17 ± 0.02^a^*	4.45 ± 0.02^b^*	2.69 ± 0.09^a^*
	12	8.55 ± 0.04^c^	6.21 ± 0.06^b^*	6.59 ± 0.02^c^*	3.59 ± 0.02^b^*
	18	8.66 ± 0.02^c^	8.30 ± 0.04^d^	8.34 ± 0.03^d^	5.36 ± 0.02^c^*
	24	9.76 ± 0.02^d^	7.51 ± 0.02^c^*	6.38 ± 0.05^c^*	5.35 ± 0.03^c^*

* All values are expressed as the mean ± standard deviation in triplicate. The lowercase letters indicate control or phage treatments, and those connected by different letters indicate a significant difference (p < 0.05). Asterisks (*) indicate significant differences (p < 0.05) between the control and treatment groups. MOI=Multiplicity of infection

In *Salmonell*a Typhimurium treatment, the phage cocktail at MOI 10^4^ reduced the count of the bacteria by 3.7 log CFU/mL at 6 h post-treatment (p < 0.05), followed by 2.3 log CFU/mL at MOI 10^2^ (p < 0.05) and 2.0 log CFU/mL at MOI 10^3^ (p < 0.05). Overall, the reduction of *S*. Typhimurium with the phage cocktail at MOI 10^4^ showed the highest reduction (4.4 log CFU/mL) at 24 h (Table 2).

The remaining phage titers were also monitored during the *in vitro* test. The phage titers were approximately 5–8 log PFU/mL for *S*. Enteritidis and 5–9 log PFU/mL for *S*. Typhimurium (Table 3).

**Table 3:** Phage titers remaining during phage treatment of representative *Salmonella* strains.

Treatments	Time (h)	Phage titers (log PFU/mL)^1^		

		MOI 10^2^	MOI 10^3^	MOI 10^4^
*Salmonella* Enteritidis	0	5.30 ± 0.13^a^	6.09 ± 0.08^a^	7.21 ± 0.04^a^
	6	5.92 ± 0.06^b^	5.98 ± 0.05^a^	7.05 ± 0.02^a^
	12	6.11 ± 0.07^b^	6.17 ± 0.06^a^	7.40 ± 0.03^a^
	18	7.54 ± 0.02^c^	7.33 ± 0.03^b^	8.11 ± 0.07^b^
	24	8.04 ± 0.04d	8.17 ± 0.06^c^	8.38 ± 0.06^b^
*Salmonella* Typhimurium	0	5.13 ± 0.05^a^	6.09 ± 0.08^a^	7.21 ± 0.04^a^
	6	6.53 ± 0.03^b^	7.44 ± 0.02^b^	7.40 ± 0.03^a^
	12	7.19 ± 0.10^b^	8.19 ± 0.07^c^	8.35 ± 0.02^b^
	18	8.37 ± 0.03^c^	8.51 ± 0.02^c^	8.57 ± 0.01^b^
	24	9.54 ± 0.02d	9.25 ± 0.01d	9.26 ± 0.03^c^

1 All values are expressed as the mean ± standard deviation in triplicate. The lowercase letters indicate phage titers, and those connected by different letters indicate a significant difference (p < 0.05). MOI=Multiplicity of infection

### Cytotoxicity of phage cocktails

**Figure 1:**
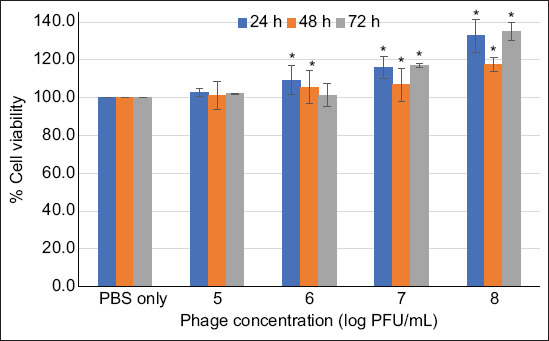
Cell viability (%) of Caco-2 cells after treatment with a phage cocktail. Asterisks (*) indicate significant differences (p < 0.05) compared with the control (PBS buffer) at each different time (24, 48, and 72 h).

To evaluate the cytotoxic effects of the developed phage cocktail, Caco-2 cells were used for the experiments. The results in Figure 1 show that the phage cocktail maintained high cell viability at >100% for all phage concentrations tested (5.0–9.0 log PFU/mL) from 24 to 72 h.

### Survivability of a phage cocktail under simulated gastrointestinal conditions

The total count of remaining phages in the cocktail under simulated gastrointestinal conditions presented 99.9% survivability (8.7 ± 0.3 log PFU/mL) in gastric fluid (pH 2.0) and 96.14± 3.45% survivability (8.4 ± 0.3 log PFU/mL) in bile salt solution. The phages, however, showed 100% (8.8 ± 0.2 log PFU/mL) in intestinal fluid (pH 8.5) compared with the phage count in a control suspension (8.8 ± 0.1 log PFU/mL).

### Survivability of phage cocktails under harsh conditions

**Figure 2:**
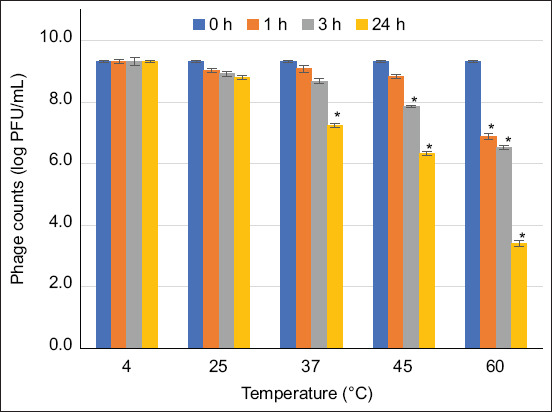
Titers of phages in cocktails survived under different temperatures. Asterisks (*) indicate significant differences (p < 0.05) compared with the control (4°C) at each different time (0, 1, 3, 24 h).

Under temperature stress, the phages maintained high survivability (74%–100%) at 4°C–60°C for 1 h, whereas a lower survivability rate was recovered at 60°C (from 9.3 ± 0.1 to 6.9 ± 0.1 log PFU/mL). At 3 and 24 h, the lowest survivability rates were 69.9% (6.5 ± 0.7 log FPU/mL) and 36.5% (3.4 ± 0.1 log PFU/mL), respectively, at 60°C. In this study, phage titers survived at 4°C and 25°C for 24 h (Figure 2).

**Figure 3:**
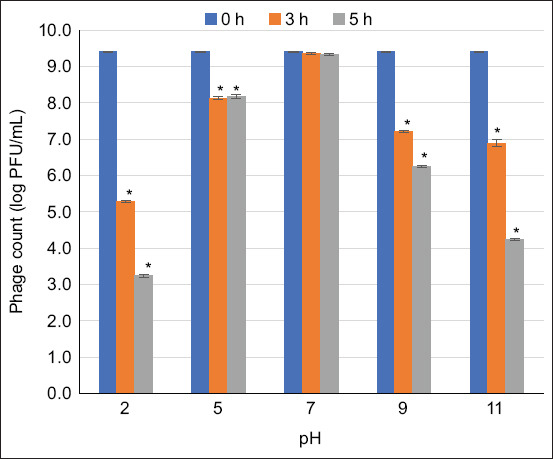
Titers of phages in cocktails survived under different pH values. Asterisks (*) indicate significant differences (p < 0.05) compared with the control (pH 7) at each different time (3 and 24 h).

**Figure 4:**
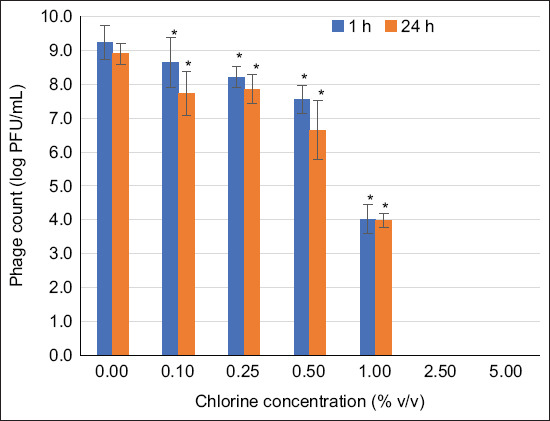
Phage titers in cocktails survived under different chlorine concentrations. The asterisk (*) indicates a significant difference (p < 0.05) compared with the control (chlorine omitted) at each time point (1 and 24 h).

Phages in a cocktail were highly stable at pH values >5, with survivability rates of 86.5% (8.1 ± 0.1 log PFU/mL) and 86.9% (8.2 ± 0.1 log PFU/mL) for 3 and 24 h, respectively (Figure 3). In addition, phage titers demonstrated over 43.1% survivability in the presence of chlorine at concentrations ranging from 0.1% to 1% (v/v). Active phages could not be recovered (0% survivability) in the presence of 2.5 and 5% (v/v) available chlorine (Figure 4).

### Survivability of phage cocktails under sanitizers

**Figure 5:**
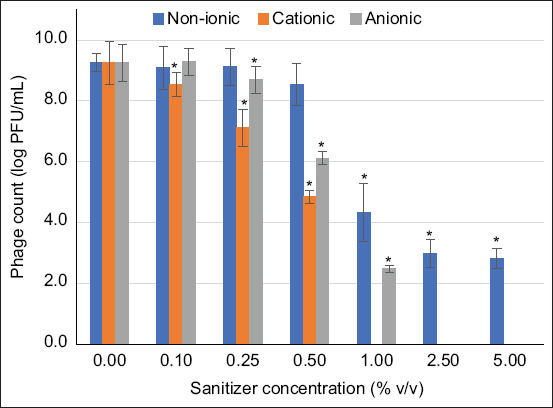
Titers of phages in cocktails survived in the presence of non-ionic, cationic, and anionic sanitizers. Asterisks (*) indicate significant difference (p < 0.05) compared with the control (sanitizers omitted).

Phage titers survived in the presence of non-ionic sanitizer at concentrations ranging from 0.1% to 5.0% (v/v), exhibiting the lowest survivability of 30.6% (2.8 ± 0.3 log PFU/mL). The phages in the cocktail showed over 52.8% survivability (4.9 ± 0.2 log PFU/mL) in the presence of cationic sanitizer at concentrations ranging from 0.1 to 0.5% (v/v). In addition, the phage cocktail was active in the presence of an anionic sanitizer at 1.0% (v/v), exhibiting 26.8% survivability (2.5 ± 0.2 log FPFU/mL) (Figure 5).

## DISCUSSION

The prevalence of *Salmonella* has been reported worldwide in the broiler supply chain (pre-harvest [farms] and post-harvest [slaughterhouses and processing plants]). Most of the infectious strains of *Salmonella* can cross-contaminate cooked food from raw food (such as chicken meat) during handling [2, 4, 5]. In this study, 251 isolates of *S. enterica* recovered from various sources of the broiler production chain were used to test the lytic ability of phages. The results showed that the phages lysed 78.2%–91.2% of the *Salmonella* isolates. This implies that the phages have a broad host range and can lyse multiple *S. enterica* isolates. However, the absence of serovar data limits our ability to fully characterize the diversity of the *Salmonella* strains included in this study. Nonetheless, these phages demonstrate a broad host range based on *Salmonella* sourced from the broiler production chain. The lytic ability recorded in this study aligns with a previous report by Pelyuntha *et al*. [15], which demonstrated that these two phages are broad-host lytic phages and is also consistent with their findings that these phages exhibit varying lytic abilities. In their study, phage WP109 exhibited 88.9% lytic activity against *Salmonella* and its multidrug-resistant strains recovered from broiler farms in the Eastern and Southern parts of Thailand, whereas phage WP128 exhibited only 77.8% lytic activity against the same groups of *Salmonella* isolates [15]. This is in contrast with the findings of Ge *et al*. [12], who reported a >96% reduction of *Salmonella* in chick feces by a *Salmonella* phage. These findings imply that the lytic activity of phages against *Salmonella* varies depending on the specific phage strain. The disparity in lytic ability among phages could be attributed to differences in bacterial receptors recognized by various phage strains, resulting in some phage strains exhibiting a narrow host range profile or serotype specificity while others demonstrate a broader range of lytic ability [17, 18]. Consequently, phages with the highest lytic activity are often recommended and employed as biocontrol agents to control *Salmonella* contamination in food production chains [14–16, 25]. The high lytic ability and broad host range of the phages in this study are advantageous, indicating that these phages could be further explored to control *Salmonella* in broiler farms and across the broiler production chain.

In general, the phage cocktail in our study had a high-efficiency level (a significant reduction of *Salmonella*) against the *Salmonella* tested, with the highest efficiency observed at MOI 10^4^. This is consistent with the study by Augustine and Barht [28], who reported significant reductions in *S*. Enteritidis in chicken cuts prepared using a phage cocktail at a higher MOI. This result could be attributed to the high number of phage particles per bacteria in treatment with an MOI of 10^4^. The MOI is the ratio of phages to bacteria in a culture; a higher MOI implies that each bacterium is exposed to multiple phages, increasing the likelihood of being infected or lysed by at least one phage and enhancing the efficiency of the phage cocktail. The high efficiency of the developed phage cocktail against the *Salmonella* isolates in this study could also be attributable to its ability to reduce the emergence of phage-resistant bacteria. This is because phage cocktails enhance bacterial receptor recognition, enabling a broader range of bacterial targets and improving the effectiveness of the cocktail. For instance, Fischer *et al*. [29] reported that a phage cocktail could reduce the resistance of bacteria to phages. The high efficiency of the phage cocktail highlights its potential as a biocontrol agent for *Salmonella* contamination in broiler production chains. The use of this agent could significantly reduce the reliance on antibiotics, which often promote antibiotic resistance, leading to treatment failures and economic losses.

Phage therapy offers significant benefits for human and animal health. This phenomenon can be attributed to the mechanism of phages, which specifically target bacterial cells by recognizing unique receptors without affecting eukaryotic cells. Consequently, phages are widely considered safe and have been designated as generally recognized as safe by the US Food and Drug Administration. In this study, the cytotoxicity of the developed phage cocktail tested on the viability of Caco-2 cells showed no trace of toxicity by the MTT assay. This aligns with the study of Wojcicki *et al*. [21], who evaluated the toxicity of the *Salmonella* phage BAFASAL^®^ (Proteon Pharmaceuticals, Poland) and reported a reduction of *S*. Enteritidis in the ceca of chickens without detecting any toxins and genotoxin or adverse effects posed by the phage, even at 100-fold doses [21]. This is also consistent with the study by Torres Di Bello *et al*. [22], who evaluated the cytotoxicity of phage Pa.7 in HaCaT cells for treating acne caused by *Cutibacterium* [22]. The findings of our study highlight the potential of the phage cocktail for application with minimal or no adverse effects, indicating that these phages could be further studied or explored for application in the broiler production chain to control *Salmonella*. However, further experiments involving live animals are needed to fully validate the safety of using this phage cocktail to control *Salmonella* infections in broilers.

The developed phage cocktail demonstrated nearly complete survivability in a simulated chicken gastrointestinal environment. The high survivability of the phage cocktail under simulated gastrointestinal conditions is consistent with the findings of Pelyuntha *et al*. [20], who reported the total survivability of the *Salmonella* phage under simulated gastrointestinal conditions in chickens. The survivability of the phage cocktail under the pH and temperature ranges reported in our study is also in agreement with previous reports by Huang *et al*. [30] and Sharma *et al*. [31], respectively. This suggests that the phage cocktail could survive in low pH conditions (1.5–3.5) in the gizzard, as well as in high pH (>8.5) in the intestine and cloaca, and it can tolerate the salt concentration in the gut. The ability of phages to withstand these conditions depends on the robustness of their capsids. Therefore, the higher the robustness of the capsid, the better its tolerance to harsh environmental conditions. The structure of a phage consists of genetic materials and a protein envelope called “capsid.” Harsh conditions (acidity, high temperature, and the presence of free-radical ions) may cause the deterioration of phage components, leading to the loss of their biological activity on targeted hosts [32]. The ability of phages to tolerate different pH levels and temperature ranges is crucial for phage administration and is essential for successful field application because these conditions often mimic the intestinal environment of animals. Acidity and alkalinity reflect the gut conditions of animals, especially when phages are applied orally. This can destroy phages with less robust capsids. The available chlorine represents the conditions encountered during phage application in the water systems of animal farms, slaughterhouses, and processing plants [33, 34]. When used in a cocktail, the phages in this study survived in all these factors, demonstrating stress-tolerant characteristics that could be beneficial for application in broiler production chains under varying conditions.

Enveloped viruses contain phospholipid bi-layered nucleocapsids that serve as target and action sites for ions, making them susceptible to detergents, surfactants, and other ionic liquids like sanitizers [35–37]. However, the phage cocktails developed in this study were highly resistant to the ionic strengths of the sanitizers applied. This is contrary to a previous study reported by Michalski *et al*. [38], the lower survivability of enveloped phages in ionic liquids. This is an advantage offered by the phage cocktail, demonstrating its ability to withstand external harsh environmental conditions during its application against *Salmonella* in the broiler production chain. This also implies that the phage cocktail could be used synergistically with sanitizers for selective or resistant bacterial (*Salmonella*) control in the broiler production chain. This is similar to previous studies that reported the potential of phages in combination with sanitizers to reduce pathogenic *Escherichia coli* strains in food, with the sanitizers having little to no effect on the phages [23, 24, 39].

In summary, the survivability of phages under simulated gastrointestinal conditions suggests that the phage cocktail holds promising potential for therapeutic applications, particularly in the treatment of *Salmonella*-induced gastrointestinal infections. Furthermore, phage resistance to sanitizers underscores their potential as effective decontamination agents in processing plants, where traditional sanitization methods may be less effective against certain pathogens. These findings support the use of phages as an innovative tool for both therapeutic and industrial applications, offering an alternative to antibiotics and traditional disinfectants for combating bacterial contamination.

## CONCLUSION

This study demonstrated the potential of a *Salmonella* phage cocktail (WP109 and WP128) as an effective biocontrol agent in the broiler production chain. The phages exhibited high lytic activity, with WP109 lysing 91.2% and WP128 lysing 78.2% of *Salmonella enterica* isolates derived from various stages of the broiler supply chain. The phage cocktail significantly reduced *Salmonella* populations *in vitro*, achieving a 4.4 log CFU/mL reduction at a multiplicity of infection of 10^4^. In addition, the cocktail maintained excellent survivability (99.9%) under simulated gastrointestinal conditions and harsh environmental factors, including low pH, temperature variations, and ionic sanitizers. Cytotoxicity testing revealed no adverse effects on Caco-2 cells, supporting the cocktail’s safety for application. The study’s strengths include the broad-spectrum lytic ability of the phage cocktail, its high stability under harsh conditions, compatibility with sanitizers, and demonstrated safety with no cytotoxic effects on host cells. However, limitations of the study include its *in vitro* design and reliance on simulated conditions, the lack of *in vivo* validation in live broilers, and incomplete serovar-level characterization of the *Salmonella* isolates. Furthermore, the cocktail’s long-term stability and efficacy in real-world storage and application conditions remain unexplored. Future research should focus on *in vivo* studies to validate the cocktail’s effectiveness and safety in broiler chickens, alongside a comprehensive characterization of *Salmonella* serovars to confirm its broad applicability. Investigations into the phage cocktail’s integration into poultry management systems, such as feed additives or water systems, would be valuable. In addition, assessing its long-term stability, efficacy in industrial-scale applications, and potential synergy with other decontamination strategies such as organic acids or modified atmosphere packaging will further support its practical implementation. This study provides a promising foundation for the application of phage cocktails as a sustainable and effective alternative to antibiotics in controlling *Salmonella* in poultry production, addressing critical challenges in food safety and public health.

## AUTHORS’ CONTRIBUTIONS

WP: Conceptualized and designed the study, conducted the experiments, data analysis, and drafted the manuscript. TN: Conducted the experiments and data analysis. DYY: Drafted the manuscript. PK: Conducted the experiments and data analysis. AS, AK, and KV: Conceptualized and designed the study. All authors have read and approved the final manuscript.
